# Cardiorespiratory Markers of Type 2 Diabetes: Machine Learning–Based Analysis

**DOI:** 10.2196/82084

**Published:** 2026-02-23

**Authors:** Flavia Maria G S A Oliveira, Sandro Muniz Cavalcanti, Michael C K Khoo

**Affiliations:** 1 Department of Electrical Engineering, School of Technology, University of Brasilia, Campus Universitário Darcy Ribeiro, Asa Norte, Brasilia-DF, 70910-900, Brazil, 55 61 3107 5510; 2 Department of Biomedical Engineering, Viterbi School of Engineering, University of Southern California, Los Angeles, CA, United States

**Keywords:** heart rate variability, frequency response function, impulse response, type 2 diabetes, diabetic autonomic neuropathy, machine learning, cardiorespiratory coupling

## Abstract

**Background:**

The global prevalence of type 2 diabetes mellitus (T2DM) poses significant challenges due to its association with increased cardiovascular risk and complications like cardiovascular autonomic neuropathy. Measures derived from heart rate variability (HRV) and cardiorespiratory interactions quantified through frequency response function (FRF) and impulse response (IR) metrics reflect different aspects of autonomic regulation and may provide complementary physiological information relevant to diabetes-related autonomic alterations.

**Objective:**

The study aimed to investigate whether these metrics, individually or in combination, provide useful physiological features for distinguishing individuals with and without T2DM using machine learning classifiers.

**Methods:**

Electrocardiogram and respiratory signals from 2 PhysioNet datasets were used to derive 3 domains of autonomic and cardiorespiratory features: (1) spectral HRV indices reflecting overall variability; (2) FRF metrics characterizing frequency-specific respiratory-cardiac transfer properties; and (3) causal IR metrics capturing time-domain responsiveness to respiratory inputs. ML classifiers—logistic regression, support vector machine (SVM) with linear kernel, and SVM with radial basis function (SVM RBF) kernel—assessed the predictive value of individual and combined feature sets under NearMiss-1 (NM) undersampling and Synthetic Minority Oversampling Technique oversampling. This systems-based framework may capture subtle differences in respiratory-cardiac regulation associated with T2DM more effectively than HRV alone by reflecting integrated cardiorespiratory coupling.

**Results:**

Across classifiers and balancing strategies, IR features frequently produced comparatively strong standalone performance, suggesting that causal, time-domain cardiorespiratory dynamics capture informative physiological differences between groups. With logistic regression and NM, IR features achieved mean accuracy of 0.770 (SD 0.179), precision of 0.783 (SD 0.217), recall of 0.900 (SD 0.224), and *F*_1_-score of 0.798 (SD 0.140). While HRV metrics were the least informative standalone feature set, the combined HRV+FRF feature set under NM yielded the highest observed performance, with accuracy of 0.830 (SD 0.172), precision of 0.800 (SD 0.183), recall of 0.933 (SD 0.149), and *F*_1_-score of 0.853 (SD 0.145; SVM RBF). Under Synthetic Minority Oversampling Technique, HRV+IR showed the strongest observed combined performance, yielding accuracy of 0.700 (SD 0.128), precision of 0.783 (SD 0.217), recall of 0.683 (SD 0.207), and *F*_1_-score of 0.691 (SD 0.097) with SVM RBF, surpassing standalone IR in most metrics, though IR alone retained superior recall (0.950, SD 0.112) and *F*_1_-score (0.708, SD 0.038). These results reflect that performance depends on both feature domain and sampling strategy and that combining features capturing complementary physiological aspects of autonomic regulation may enhance discriminative ability.

**Conclusions:**

HRV, FRF, and IR metrics each reflect distinct dimensions of autonomic and cardiorespiratory regulation. Systems-based approaches incorporating frequency-domain and causal dynamic features may offer richer characterization of diabetes-related regulatory differences than HRV alone. Although preliminary and limited by sample size, these findings highlight promising physiological feature domains and sampling strategies for future investigation. Larger datasets with well-defined autonomic phenotyping are needed to evaluate generalizability and determine clinical relevance.

## Introduction

The International Diabetes Federation [1] reports that global prevalence of diabetes in adults aged 20‐79 years rose from 151 million (about 4.6% of the global population) in 2000, to 589 million (11.1%) in 2024, with projections reaching 853 million (13.0%) by 2050. Over 90% of these cases are type 2 diabetes mellitus (T2DM). Diabetes is associated with a twofold increase in the risk of vascular diseases, including coronary heart disease and stroke, independent of common risk factors such as obesity and hypertension [2].

Cardiovascular autonomic neuropathy (CAN), affecting from 12% to 73% of patients with T2DM and linked to a 3.45-fold mortality risk [3], is a highly prevalent but frequently overlooked microvascular complication, also found in prediabetic individuals with metabolic syndrome [4]. Sörensen et al [5] demonstrated that generalized microvascular dysfunction is already present in prediabetes and becomes more pronounced in established T2DM. These findings suggest that microvascular impairment precedes and may contribute to later cardiovascular complications in T2DM, supporting its role as a potential early target for intervention.

Reduced heart rate variability (HRV) is an early sign of CAN in individuals with diabetes and prediabetes [6]. HRV has been used in many studies to quantify cardiovascular autonomic function in T2DM, demonstrating that diabetes significantly affects both the sympathetic and parasympathetic branches of the autonomic nervous system [7]. Lower HRV in diabetic subjects, compared to controls, indicates an impaired ability to adapt to physiological stressors, such as physical exercise and orthostatic stress. Using HRV to measure cardiac autonomic function, Wang et al [8] demonstrated that autonomic dysfunction precedes the development of T2DM, particularly in younger individuals, even after adjusting for cardiovascular risk factors. Given that HRV metrics can capture changes in autonomic control associated with diabetes progression [9], they may provide insight into early physiological alterations in individuals at risk for T2DM. Some studies have advocated the use of standardized cardiovascular autonomic reflex tests (CARTs) rather than measurements of spontaneous HRV, but a major limitation of CARTs is that they require varying levels of subject cooperation, and differing levels of attention or anxiety may lead to intersubject and intrasubject variability in test results [10].

While HRV analysis offers important insights into cardiovascular autonomic regulation, it is well known that respiratory patterns influence HRV (eg, respiratory sinus arrhythmia), potentially confounding cardiovascular autonomic assessments. Different approaches have been proposed in the literature to address this issue. One approach is to estimate the frequency response function (FRF), or spectral transfer function, between changes in respiration (as input) to variations in R-to-R interval (RRI) (as output) [11-13]. For instance, Khoo et al [11] demonstrated that the average transfer gain between respiration and RRI, an FRF-derived metric representing vagal control that specifically accounts for respiratory influences, outperformed traditional HRV by explicitly accounting for respiratory contributions. In the context of T2DM, such FRF-based descriptors may offer complementary insights into cardiorespiratory interactions that differ between individuals with and without diabetes, highlighting physiological dimensions not captured by HRV alone.

Since the cardiovascular and respiratory systems are inherently interconnected in a closed-loop framework, a limitation of the FRF approach is its assumption of a 1-directional, feedforward influence from input to output, with no reciprocal feedback. However, these systems interact bidirectionally, with cardiovascular and respiratory signals influencing each other. This simplification may complicate the interpretation of results. To address the inherent noncausal nature of the FRF, an alternative approach is to estimate the time-domain impulse response (IR) between measured input and output within a mathematical model of the underlying dynamics. This representation allows the output to depend explicitly on present and past values of the input, but not future values, as well as the inclusion of delays into the model. This effectively “opens the loop,” helping separate feedforward influences from feedback interactions [14,15].

In this study, we sought to investigate how these different, complementary measures of autonomic regulation—spectral measures of HRV, noncausal systems-based FRF metrics, and causal IR-derived indices—differ between subjects with and without T2DM. We then examined whether these physiologically grounded descriptors provide discriminative value for distinguishing T2DM from controls in machine learning (ML) models.

To the best of our knowledge, this is the first study to evaluate HRV, FRF, and IR metrics collectively in a ML framework to examine physiologically grounded differences between individuals with and without T2DM. By systematically evaluating individual and combined feature domains, this study provides exploratory evidence on the potential value of multivariate, systems-based physiological descriptors in distinguishing diabetes-related regulatory patterns.

## Methods

### Database

This study used 2 publicly available PhysioNet repositories curated by the same research group: the cerebral vasoregulation in diabetes dataset [16] and the cerebral perfusion and cognitive decline in type 2 diabetes dataset [17]. Both datasets were collected at the Syncope and Falls in the Elderly Laboratory at Beth Israel Deaconess Medical Center (BIDMC), Harvard Medical School, Boston, MA, by the research group of Dr. Vera Novak, under comparable experimental conditions and using the same core infrastructure, ensuring methodological compatibility. Among other measures, the studies analyzed electrocardiogram (ECG) and respiration signals recorded during a standardized “sit-to-stand” test in subjects with T2DM and age-matched controls, aged 50‐85 years.

The demographics and clinical characteristics, including hemoglobin A1c, of the groups that participated in the sit-to-stand test in both datasets are summarized in Table 1, reflecting mean and SD of available data, as some participants lacked demographic (n=2) or clinical (n=8) information.

**Table 1.** Main characteristics of the groups.

Variable	Groups, mean (SD)		*P* value
	Control (n=21)	T2DM^a^ (n=49)	
Age (y)	64.6 (8.1)	63.7 (8.1)	.61
Mass (kg)	68.6 (11.2)	83.2 (15)	<.001^b^
BMI	24.2 (2.6)	29.0 (5.1)	<.001^b^
HbA_1c_^c^ (%)	5.4 (0.4)	7.2 (1.4)	<.001^b^
RRI^d^ (ms)	877.4 (144.9)	825.8 (137.8)	<.001^b^

a T2DM: type 2 diabetes mellitus.

b *P*≤.05 (data are mean [SD]).

c HbA_1c_: hemoglobin A_1c_.

d RRI: R-to-R interval.

### Ethical Considerations

This study involved secondary analysis of two publicly available, deidentified datasets hosted on PhysioNet: the Cerebral Vasoregulation in Diabetes dataset and the Cerebral Perfusion and Cognitive Decline in Type 2 Diabetes dataset. The original data collections were conducted at BIDMC under institutional review board (IRB) approval (IRB 2003P000013 and IRB 2005P000338, respectively). All participants provided written informed consent prior to enrollment in the original studies.

In the original protocols, participants were admitted to the Clinical Research Center at BIDMC, where all study procedures were conducted under medical supervision. Privacy and confidentiality were maintained under IRB-approved procedures, and data were handled in accordance with institutional and federal regulations governing human subjects research. The datasets made available on PhysioNet were fully deidentified prior to public release, and no personally identifiable information is included.

This study used only publicly available, deidentified data and did not involve direct contact with participants. Therefore, no additional ethics approval or informed consent was required for this secondary analysis.

The original study documentation does not specify whether participants received financial compensation for participation.

All procedures adhered to the ethical standards of the responsible IRBs and to the principles outlined in the Declaration of Helsinki.

### Data Preprocessing

For accurate interpretation of spectral measures of HRV, the data should be essentially stationary. As per the European Society of Cardiology/North American Society of Pacing and Electrophysiology Task Force on HRV [18], recordings should be short enough to meet stationarity requirements for frequency-domain analyses, yet long enough to capture at least 10 cycles of the cut-off frequency for the low-frequency (LF) HRV component, typically set at 0.04 Hz (corresponding to a periodicity of 25 s). Taking these considerations into account, we selected 4-minute (240 s) segments in the sitting position for analysis. Recordings with significant signal loss due to equipment recalibration, brief unexplained signal flattening (repeatedly found in the respiratory signals), or excessive ectopic beats were excluded from analysis. In the recordings that were available for this study, only about half of the participants had data measured in both sitting and standing postures. To maximize sample size for our analyses, we chose to use data from the sitting posture only.

The original pooled dataset included 21 control subjects and 49 individuals with T2DM. However, because the IR feature set requires each subject to have both valid ECG and respiration signals, we restricted analysis to subjects who had valid recordings of both signals. To ensure directly comparable evaluations of all feature sets (HRV, FRF, and IR), we elected to use the same subset of participants across every machine-learning analysis. After applying all preprocessing steps, the final consistent dataset comprised 18 T2DM subjects and 11 controls. These fixed sample sizes were used for all classifiers and all feature-set comparisons to avoid bias introduced by varying subject availability across methods.

Data processing was performed using the Cardiorespiratory System Identification Lab [19], a freely available MATLAB-based software tool for evaluating autonomic nervous system function through HRV and cardiorespiratory system analysis. Key steps included detecting R-waves in the ECG using a Pan-Tompkins-based algorithm [20] to obtain the RRI time series and converting the airflow data (in mL/s) into instantaneous lung volume (ILV) in mL. To prepare the data for spectral and IR analyses, the RRI and ILV signals were resampled at 4 Hz after detrending to eliminate very LF oscillations [21].

### Spectral Analysis of HRV

Spectral analysis of the resampled HRV signal was conducted using power spectral density estimation via the Welch method with a Hann window to minimize spectral leakage. We calculated the LF (0.04 to 0.15 Hz) and high-frequency (HF) (0.15 to 0.4 Hz) [18] components of the RRI time series, as well as the LF by HF ratio. These spectral indices provide insights into cardiac autonomic modulation, with $HRV_{HF}$ often linked to vagal activity and $HRV_{LF}$ reflecting a mix of sympathetic and parasympathetic inputs [18,22-24], with the ratio $HRV_{LF/HF}\left(HRV_{LF}/HRV_{HF}\right)$ commonly interpreted as a measure of “sympathovagal balance,” although this view has been challenged [25-27].

### System-Based Analyses in the Frequency and Time Domains

To incorporate the influence of respiratory-heart rate coupling, we used the FRF and IR analyses. FRF estimates how an output response (eg, RRI) is modulated by an input (eg, respiration), providing a frequency-based perspective on autonomic regulation [28]. Specifically, the FRF gain quantifies the efficiency of coupling between respiratory inputs and cardiac responses, with higher gains indicating stronger modulation of heart rate by respiratory oscillations [28]. In this study, we calculated FRF gain values for LF and HF bands (${\left|FRF\right|}_{LF}$ and ${\left|FRF\right|}_{HF}$, respectively) to quantify respiratory influences on RRI. By analyzing frequency-specific dynamics, the FRF highlights how respiratory-cardiac coupling (RCC) varies across physiologically relevant frequency bands. However, FRF is inherently limited in its ability to assess causal interactions, as it does not disentangle feedforward from feedback mechanisms or establish directionality [29].

To address these limitations, IR analysis was used to provide a time-domain, causal perspective on the system’s dynamics. By modeling the system’s response to an impulse input in respiration, IR analysis allows for the characterization of the system’s adaptability, assessing how effectively and over what time frame the cardiovascular system can adjust to respiratory inputs or other perturbations.

To quantify the ILV-to-RRI IR, we calculated several key descriptors: IR magnitude, which reflects the strength of the immediate respiratory influence on cardiac output; dynamic gain (DG, using total, LF, and HF components), which represents the average magnitude of the system’s influence across different frequency bands; and characteristic time (*t*_char_), which captures the time it takes for the response to occur or subside, providing insights into delayed or sustained effects.

These descriptors facilitate statistical comparisons between groups and capture essential regulatory characteristics of the RCC mechanism [15]. Together, these metrics help reveal the system’s ability to maintain stability and recover from changes, offering critical insights into the system’s flexibility and robustness in both health and disease [15,30]. Further details on the FRF and IR methodologies are available in the online supplementary material (Multimedia Appendix 1).

### Analysis Procedures

#### Machine Learning Classifiers

To distinguish T2DM subjects from controls, we used three ML classifiers: logistic regression (LR), support vector machines (SVMs) with linear kernels (SVM linear), and SVM with radial basis function (SVM RBF kernels). We trained and tested these classifiers using various feature sets—as described below—which capture distinct but complementary aspects of autonomic nervous system function.

#### Feature Sets and Groupings

Although HRV, FRF, and IR features all originate from the cardiac timing signal (RRI), they differ in the extent to which they incorporate respiratory information and in the physiological mechanisms they reflect. Spectral HRV indices (LF, HF, and LF/HF) quantify the distribution of oscillatory RRI variability but remain univariate summary descriptors of oscillatory patterns influenced by multiple regulatory pathways. FRF metrics quantify the frequency-specific transfer characteristics of respiratory—cardiac interactions, capturing the gain and phase relationships that reflect how respiratory oscillations shape cardiac timing, but without modeling causal direction. IR metrics extend this systems-based perspective by characterizing the causal, time-domain responsiveness of RRI to respiratory perturbations, thereby providing information about dynamic adaptability and directional regulation that is not accessible from HRV or FRF measures alone. Given these complementary perspectives—overall variability patterns (HRV), frequency-dependent transfer behavior (FRF), and causal dynamics responsiveness (IR)—we additionally evaluated whether combining HRV with FRF or IR features provided complementary discriminative information beyond any single feature domain.

Thus, initially, each classification model was trained on one of the following individual feature sets: (a) HRV metrics, (b) FRF metrics, or (c) IR metrics. To assess whether combining feature sets could enhance classification performance, we created additional feature groupings: (d) HRV+FRF metrics and (e) HRV+IR metrics.

Covariates, including BMI, were not incorporated into the ML models. The present study was designed to examine the physiological content and discriminative behavior of HRV, FRF, and IR features, rather than to develop covariate-adjusted predictive models. This approach reflects the mechanistic focus of this study and avoids the added model complexity and potential instability that covariate adjustment would introduce given the modest sample size.

#### Handling Class Imbalance

To address the T2DM majority (accounting for about 2/3 of subjects), we applied NearMiss-1 (NM) undersampling [31] and Synthetic Minority Oversampling Technique (SMOTE) [32], comparing performance against the unbalanced dataset. This approach allowed us to evaluate classifier performance under different class balance conditions and to compare the relative effectiveness of undersampling and oversampling in improving predictive accuracy. A balanced training dataset in ML is commonly used to reduce bias toward the majority class.

In this exploratory study, NM and SMOTE were applied to the full usable dataset prior to generating the 5-fold stratified cross-validation partitions, rather than separately within each training fold. This design allowed for direct comparison of balancing strategies under fixed class distributions, but it also entails that, particularly for SMOTE, synthetic samples were generated using neighborhood information from the entire dataset. As a result, some synthetic samples may appear in both training and test folds. The resulting performance estimates should therefore be viewed within the exploratory, hypothesis-generating scope of the study.

With the usable dataset consisting of 18 T2DM and 11 control subjects, NM undersampling reduced the majority class to match the minority class, yielding 11 T2DM and 11 control subjects (N=22 total). In contrast, SMOTE oversampling synthesized 7 new control samples, producing a balanced dataset of 18 T2DM and 18 controls (N=36 total). All classification models under each balancing strategy were trained using these corresponding sample sizes.

#### Data Preprocessing and Feature Standardization

Given that features in our dataset span different units, all features were standardized using *z*-score normalization (mean 0, SD 1). This preprocessing step ensures that features with larger values do not dominate the model, which is particularly relevant for distance-based methods like SVM [33]. Standardizing features also improves interpretability in LR, as larger coefficients indicate higher feature importance in classification [34,35].

As with the resampling procedures, *z*-score normalization was applied once, before cross-validation, to maintain consistent feature scaling across all model comparisons. This approach trades off strict fold-wise isolation for stability in a small dataset.

#### Cross-Validation and Feature Correlation

Prior to model training, we performed correlation analysis within each feature set to identify and exclude features with correlations above 0.8. This minimized redundancy and mitigated multicollinearity, promoting stable classification model estimates and clearer interpretation of feature contributions [36,37]. Applying this filtering prior to cross-validation ensured consistent feature definitions across classifiers and balancing strategies, which would not have been feasible with fold-wise filtering given the modest sample size. Models were then evaluated using 5-fold stratified cross-validation to obtain a more robust estimate of classification performance and reduce the risk of overfitting [38].

#### Performance Metrics and Evaluation

Each classifier’s performance was assessed via accuracy, precision, sensitivity, specificity, *F*_1_-score, and area under the receiver operating characteristic curve (AUC-ROC), averaged across 5-fold cross-validation. Each metric offers unique insights into different aspects of classifier performance:

- Accuracy measures the overall correctness of the classifier by calculating the proportion of correctly classified instances (both true positives and true negatives) among all instances; however, it may be misleading in imbalanced datasets, where the majority class dominates the metric.
- Precision evaluates the proportion of true positive predictions among all positive predictions (true positives + false positives), highlighting the classifier’s ability to avoid false positives.
- Sensitivity (recall) reflects the model’s capacity to identify true positives among all actual positives (true positives + false negatives), a key metric in clinical contexts where missing true positive cases (false negatives) can be costly.
- Specificity assesses the model’s performance in correctly identifying true negatives among all actual negatives (true negatives + false positives), important for determining how well the classifier avoids false positives.
- *F*_1_-score is the harmonic mean of precision and sensitivity, providing a single balanced metric that is useful when classes are imbalanced.
- AUC-ROC summarizes the trade-off between sensitivity (true positive rate) and 1−specificity (false positive rate) across different decision thresholds, indicating the classifier’s ability to distinguish between classes, with a higher AUC-ROC value reflecting better overall performance.

Considering multiple performance metrics provides a comprehensive assessment of each classifier. This approach reveals strengths and limitations that may not be apparent if relying solely on accuracy, especially for unbalanced datasets, which are common in biomedical data. In this study, we evaluated these multiple performance metrics to assess each classifier’s performance on imbalanced, undersampled, and oversampled datasets. We aimed to identify the technique that most effectively mitigated class imbalance effects and enhanced model robustness.

Given the modest sample size and the exploratory nature of the analysis, performance comparisons across classifiers, feature sets, and balancing strategies were evaluated descriptively using cross-validated metrics to highlight general performance patterns. These observed differences provide useful preliminary indications of how different models and feature sets behave under the tested conditions.

## Results

The results presented provide insight into how each feature set and sampling method influenced the performance for the T2DM versus control classification task.

### Classification Performance With Individual Feature Sets Using the Full (Unbalanced) Dataset

Table 2 shows a comparison of the performance metrics for individual features using the full (unbalanced) dataset, for all classifiers (LR, SVM with linear kernel, and SVM with RBF kernel).

**Table 2.** Classification performance using unbalanced data (all samples)^a^.

Feature set and classifier, mean (SD)	Accuracy, mean (SD)	Precision, mean (SD)	Recall, mean (SD)	Specificity, mean (SD)	*F*_1_-score, mean (SD)	AUC-ROC^b^, mean (SD)
HRV^c^						
LR^d^	0.487 (0.152)	0.553 (0.077)	0.750 (0.354)	0.100 (0.224)	0.610 (0.192)	0.525 (0.130)
SVM^e^ linear	0.587 (0.084)	0.620 (0.073)	0.900 (0.224)	0.100 (0.224)	0.718 (0.098)	0.428 (0.208)
SVM RBF^f^	0.587 (0.084)	0.620 (0.073)	0.900 (0.224)	0.100 (0.224)	0.718 (0.098)	0.617 (0.311)
FRF^g^						
LR	0.613 (0.168)	0.627 (0.128)	0.800 (0.298)	0.233 (0.325)	0.692 (0.201)	0.606 (0.268)
SVM linear	0.620 (0.073)	0.620 (0.073)	0.933 (0.149)	0.067 (0.149)	0.744 (0.099)	0.533 (0.326)
SVM RBF	0.620 (0.073)	0.620 (0.073)	0.933 (0.149)	0.067 (0.149)	0.744 (0.099)	0.464 (0.137)
IR^h^						
LR	0.660 (0.106)	0.700 (0.183)	0.933 (0.149)	0.200 (0.447)	0.773 (0.060)	0.372 (0.205)
SVM linear	0.620 (0.073)	0.633 (0.075)	0.933 (0.149)	0.100 (0.224)	0.747 (0.073)	0.575 (0.205)
SVM RBF	0.620 (0.073)	0.620 (0.073)	1.000 (0.000)	0.000 (0.000)	0.763 (0.058)	0.700 (0.126)

a Performance metrics using individual features (HRV, FRF, or IR) for each classification model (LR, SVM with linear kernel, and SVM with RBF kernel) using the full, unbalanced dataset.

b AUC-ROC: area under the receiver operating characteristic curve.

c HRV: heart rate variability.

d LR: logistic regression.

e SVM: support vector machine.

f RBF: radial basis function.

g FRF: frequency response function.

h IR: impulse response.

Upon analyzing the classification performance metrics obtained for the individual feature sets—HRV, FRF, and IR metrics—we observed notable differences. Using the unbalanced dataset, the IR feature set yielded the highest metric values among the 3 domains, with accuracy (0.660, SD 0.106; LR), precision (0.700, SD 0.183; LR), recall (0.933, SD 0.149; LR and SVM linear), and *F*_1_-score (0.773, SD 0.060; LR). FRF showed intermediate performance, while HRV consistently exhibited the lowest values across classifiers.

A comparative analysis of model performance indicated that the SVM linear and SVM RBF models performed comparably to the LR model across most metrics. For the IR feature set, the SVM RBF model achieved the highest recall (1.000, SD 0.000), while both LR and SVM linear classifiers had a recall of 0.933 (SD 0.149). The LR classifier exhibited the highest precision (0.700, SD 0.183) and *F*_1_-score (0.773, SD 0.060).

Specificity was low across all feature sets in the unbalanced dataset, in which approximately two-thirds of the subjects are T2DM, hindering accurate classification of negative samples. This underscores the importance of using appropriate strategies to address data imbalance for improved model performance.

### Impact of NearMiss-1 Undersampling on Individual Feature Sets

Tables3  4 show a comparison of performance metrics for individual features using NM undersampling and SMOTE oversampling, respectively. NM improved performance across all feature sets. The IR feature often demonstrated comparatively stronger performance (eg, LR: accuracy 0.770, SD 0.179; precision 0.783, SD 0.217; recall 0.900, SD 0.224; specificity 0.633, SD 0.415; and *F*_1_-score 0.798, SD 0.140), exceeding both HRV and FRF features in several metrics. While FRF features performed well, particularly in recall (1.000, SD 0.00) for both SVM linear and SVM RBF models, the IR features provided more balanced performance across all metrics. The HRV feature set consistently exhibited the lowest performance metrics in this scenario.

**Table 3.** Comparison of performance metrics across feature sets and classifiers using NearMiss-1 (NM) undersampling (individual features)^a^.

Feature set and classifier	Accuracy, mean (SD)	Precision, mean (SD)	Recall, mean (SD)	Specificity, mean (SD)	*F*_1_-score, mean (SD)	AUC-ROC^b^, mean (SD)
HRV^c^						
LR^d^	0.640 (0.272)	0.567 (0.365)	0.733 (0.435)	0.567 (0.253)	0.783 (0.158)	0.767 (0.224)
SVM^e^ linear	0.730 (0.192)	0.683 (0.207)	0.900 (0.224)	0.567 (0.253)	0.765 (0.190)	0.767 (0.224)
SVM RBF^f^	0.730 (0.192)	0.683 (0.207)	0.900 (0.224)	0.567 (0.253)	0.765 (0.190)	0.767 (0.224)
FRF^g^						
LR	0.560 (0.251)	0.580 (0.239)	0.767 (0.325)	0.400 (0.418)	0.628 (0.230)	0.533 (0.298)
SVM linear	0.640 (0.251)	0.630 (0.244)	1.000 (0.000)	0.300 (0.447)	0.752 (0.173)	0.667 (0.204)
SVM RBF	0.640 (0.251)	0.630 (0.244)	1.000 (0.000)	0.300 (0.447)	0.752 (0.173)	0.500 (0.373)
IR^h^						
LR	0.770 (0.179)	0.783 (0.217)	0.900 (0.224)	0.633 (0.415)	0.798 (0.140)	0.700 (0.447)
SVM linear	0.600 (0.235)	0.600 (0.235)	1.000 (0.000)	0.200 (0.447)	0.731 (0.163)	0.767 (0.253)
SVM RBF	0.540 (0.185)	0.525 (0.145)	0.700 (0.447)	0.367 (0.415)	0.654 (0.178)	0.733 (0.308)

a Performance metrics of balanced datasets using individual features (HRV, FRF, or IR), by applying NM undersampling.

b AUC-ROC: area under the receiver operating characteristic curve.

c HRV: heart rate variability.

d LR: logistic regression.

e SVM: support vector machine.

f RBF: radial basis function.

g FRF: frequency response function.

h IR: impulse response.

**Table 4.** Comparison performance metrics across feature sets and classifiers using Synthetic Minority Oversampling Technique (SMOTE)–balanced data (individual feature sets)^a^.

Feature set and classifier	Accuracy, mean (SD)	Precision, mean (SD)	Recall, mean (SD)	Specificity, mean (SD)	*F*_1_-score, mean (SD)	AUC-ROC^b^, mean (SD)
HRV^c^						
LR^d^	0.550 (0.167)	0.619 (0.230)	0.617 (0.361)	0.500 (0.373)	0.547 (0.171)	0.654 (0.169)
SVM^e^ linear	0.607 (0.164)	0.586 (0.114)	0.833 (0.236)	0.383 (0.274)	0.673 (0.130)	0.717 (0.162)
SVM RBF^f^	0.604 (0.192)	0.686 (0.288)	0.683 (0.207)	0.567 (0.435)	0.639 (0.136)	0.683 (0.190)
FRF^g^						
LR	0.446 (0.215)	0.450 (0.132)	0.633 (0.280)	0.250 (0.250)	0.523 (0.187)	0.408 (0.264)
SVM linear	0.446 (0.074)	0.380 (0.217)	0.633 (0.375)	0.250 (0.306)	0.592 (0.069)	0.300 (0.326)
SVM RBF	0.450 (0.133)	0.394 (0.229)	0.683 (0.410)	0.200 (0.209)	0.623 (0.097)	0.233 (0.320)
IR^h^						
LR	0.529 (0.156)	0.587 (0.250)	0.600 (0.379)	0.517 (0.291)	0.520 (0.190)	0.629 (0.193)
SVM linear	0.611 (0.062)	0.569 (0.041)	0.950 (0.112)	0.267 (0.181)	0.708 (0.038)	0.771 (0.062)
SVM RBF	0.532 (0.088)	0.434 (0.247)	0.617 (0.439)	0.417 (0.328)	0.615 (0.146)	0.692 (0.216)

a Performance metrics of balanced datasets using individual features (HRV, FRF, or IR), by applying SMOTE oversampling.

b AUC-ROC: area under the receiver operating characteristic curve.

c HRV: heart rate variability.

d LR: logistic regression.

e SVM: support vector machine.

f RBF: radial basis function.

g FRF: frequency response function.

h IR: impulse response.

Notably, applying NM undersampling improved specificity across all classification models (eg, LR: increased from 0.100, SD 0.224 to 0.567, SD 0.253), enhancing its reliability in classifying negative samples (ie, control subjects) while reducing false positives.

The SVM linear and SVM RBF models tended to show higher accuracy and recall than the LR classifier for the HRV and FRF feature sets, although their *F*_1_-scores were not consistently higher. For the IR feature set, the LR classifier generally produced metrics that were equal to or slightly higher than those of the SVM models. The 2 SVM classifiers exhibited very similar performance metrics across each individual feature set, with the RBF kernel showing slight advantages in certain cases. Overall, the IR feature set paired with the LR model showed some of the comparatively stronger results on the balanced dataset.

### Classification Performance Using SMOTE Oversampling on Individual Feature Sets

SMOTE oversampling enhanced performance across all feature sets compared to the unbalanced dataset. Within this setting, IR features frequently showed comparatively strong performance across classifiers, with the LR model showing the highest precision (0.587, SD 0.250) and specificity (0.517, SD 0.291) among all classifiers. The SVM linear classifier achieved the highest accuracy (0.611, SD 0.062), recall (0.950, SD 0.112), *F*_1_-score (0.708, SD 0.038), and AUC-ROC (0.771, SD 0.062), while the highest specificity was obtained from the LR model (0.517, SD 0.291).

The HRV features produced the next highest metric values, with the SVM RBF model showing the highest precision (0.686, SD 0.288) and specificity (0.567, SD 0.435). Both SVM models showed comparable *F*_1_-score (0.673, SD 0.130 for SVM linear) and AUC-ROC (0.717, SD 0.162 for SVM linear). While the LR model benefited from SMOTE balancing compared to the original dataset, it showed slightly lower overall metrics relative to the SVM models.

The FRF feature set, however, showed lower overall performance across all classifiers compared to HRV and IR, indicating limited discriminatory power, particularly in specificity and precision. This suggests that the FRF indices may lack comprehensive information required for effective classification across all classifiers when using the SMOTE-oversampled dataset.

In terms of model comparisons, the SVM linear classifier generally showed comparatively strong performance across feature sets, particularly when paired with IR metrics. The SVM RBF model demonstrated high recall but tended to have lower precision and *F*_1_-scores than SVM linear. Although SMOTE improved LR performance, it lagged behind SVM models, except when using the IR feature set, where it showed competitive results.

Overall, SMOTE balancing improved specificity and overall classification reliability over the unbalanced dataset. The combination of IR features with the SVM linear model produced some of the strongest performance patterns observed in this analysis, underscoring how feature-classifier interactions can influence discrimination. These patterns offer hypothesis-generating observations that warrant evaluation in larger datasets.

### Comparative Effectiveness of Nearmiss-1 and SMOTE for Classifier Performance on Individual Feature Sets

Comparing the performance metrics of the NM and SMOTE data balancing approaches, both techniques clearly enhanced classification metrics over the unbalanced dataset but had different strengths. NM undersampling often led to higher specificity across classifiers, indicating better identification of negative samples. This was particularly notable with the IR feature set, where the LR (NM) model achieved higher recall (0.900, SD 0.224) and a superior *F*_1_-score (0.798, SD 0.140) compared to LR (SMOTE), which showed lower recall (0.600, SD 0.379) and *F*_1_-score (0.520, SD 0.19).

The recall and *F*_1_-scores of the SVM linear model using balanced data from either NM or SMOTE strategies were mostly similar, suggesting comparable performance of the two balancing methods for this classifier. While SVM RBF (NM) showed slightly better recall and *F*_1_-score than SVM RBF (SMOTE), the differences were marginal due to the high variability in the results.

In general, SMOTE effectively enhanced sensitivity, well-suited for identifying T2DM cases, though its effectiveness varied by classifier and feature set, with inconsistent AUC-ROC gains.

NM, on the other hand, was more effective for improving specificity and *F*_1_-scores (eg, LR and SVM linear with IR), particularly advantageous for non-T2DM classification. These results underscore the importance of selecting the appropriate data balancing approach based on specific classification goals and the clinical implications of false positives versus false negatives.

### Combined Feature Sets Analysis

We also evaluated whether combining HRV, FRF, and IR features would enhance classification performance. Table 5 shows a comparison of the performance metrics for the combined HRV+FRF and HRV+IR feature sets, along with the metrics of the individual IR feature set, using the full (unbalanced) dataset, for all classifiers (LR, SVM linear, and SVM RBF).

For the full, unbalanced dataset, combinations (HRV+FRF and HRV+IR) did not provide a significant advantage over using individual FRF or IR feature sets. The IR feature set alone generally showed the strongest performance patterns across models, with comparatively higher accuracy, precision, recall, and *F*_1_-score relative to the combined feature sets in this setting.

Tables6  7 show a comparison of performance metrics for the combined HRV+FRF and HRV+IR feature sets, along with those for the individual IR feature set, using NM undersampling and SMOTE oversampling, respectively.

**Table 5.** Comparison of performance metrics for combined feature sets (HRV^a^+FRF^b^, HRV+IR^c^) and individual lR feature set using unbalanced data^d^.

Feature set and classifier	Accuracy, mean (SD)	Precision, mean (SD)	Recall, mean (SD)	Specificity, mean (SD)	*F*_1_-score, mean (SD)	AUC-ROC^e^, mean (SD)
HRV+FRF						
LR^f^	0.553 (0.141)	0.520 (0.292)	0.700 (0.447)	0.233 (0.325)	0.730 (0.109)	0.469 (0.291)
SVM^g^ linear	0.620 (0.182)	0.613 (0.173)	0.867 (0.298)	0.167 (0.236)	0.714 (0.219)	0.422 (0.248)
SVM RBF^h^	0.547 (0.166)	0.567 (0.149)	0.800 (0.298)	0.067 (0.149)	0.661 (0.208)	0.656 (0.374)
HRV+IR						
LR	0.553 (0.141)	0.600 (0.091)	0.833 (0.236)	0.100 (0.224)	0.687 (0.124)	0.467 (0.302)
SVM linear	0.587 (0.084)	0.620 (0.073)	0.883 (0.162)	0.100 (0.224)	0.720 (0.073)	0.492 (0.298)
SVM RBF	0.547 (0.117)	0.587 (0.084)	0.883 (0.162)	0.000 (0.000)	0.701 (0.098)	0.597 (0.284)
IR						
LR	0.660 (0.106)	0.700 (0.183)	0.933 (0.149)	0.200 (0.447)	0.773 (0.060)	0.372 (0.205)
SVM linear	0.620 (0.073)	0.633 (0.075)	0.933 (0.149)	0.100 (0.224)	0.747 (0.073)	0.575 (0.205)
SVM RBF	0.620 (0.073)	0.620 (0.073)	1.000 (0.000)	0.000 (0.000)	0.763 (0.058)	0.700 (0.126)

a HRV: heart rate variability.

b FRF: frequency response function.

c IR: impulse response.

d Performance metrics using combined feature sets (HRV+FRF and HRV+IR), compared to the individual IR feature set, for each classification model (LR, SVM linear, and SVM RBF) using the full, unbalanced dataset.

e AUC-ROC: area under the receiver operating characteristic curve.

f LR: logistic regression.

g SVM: support vector machine.

h RBF: radial basis function.

**Table 6.** Comparison of performance metrics for combined feature sets (HRV^a^+FRF^b^, HRV+IR^c^) and individual IR set, using NearMiss-1 (NM) balanced data^d^.

Feature set and classifier	Accuracy, mean (SD)	Precision, mean (SD)	Recall, mean (SD)	Specificity, mean (SD)	*F*_1_-score, mean (SD)	AUC-ROC^e^, mean (SD)
HRV+FRF						
LR^f^	0.680 (0.280)	0.667 (0.236)	0.833 (0.236)	0.533 (0.361)	0.733 (0.221)	0.667 (0.295)
SVM^g^ linear	0.730 (0.076)	0.667 (0.000)	0.933 (0.149)	0.533 (0.075)	0.773 (0.060)	0.767 (0.253)
SVM RBF^h^	0.830 (0.172)	0.800 (0.183)	0.933 (0.149)	0.733 (0.253)	0.853 (0.145)	0.800 (0.274)
HRV+IR						
LR	0.680 (0.344)	0.767 (0.325)	0.667 (0.312)	0.700 (0.447)	0.693 (0.300)	0.783 (0.298)
SVM linear	0.730 (0.192)	0.767 (0.224)	0.833 (0.236)	0.633 (0.415)	0.760 (0.146)	0.800 (0.274)
SVM RBF	0.680 (0.125)	0.700 (0.183)	0.833 (0.236)	0.533 (0.361)	0.720 (0.073)	0.667 (0.312)
IR						
LR	0.770 (0.179)	0.783 (0.217)	0.900 (0.224)	0.633 (0.415)	0.798 (0.140)	0.700 (0.447)
SVM linear	0.600 (0.235)	0.600 (0.235)	1.000 (0.000)	0.200 (0.447)	0.731 (0.163)	0.767 (0.253)
SVM RBF	0.540 (0.185)	0.525 (0.145)	0.700 (0.447)	0.367 (0.415)	0.654 (0.178)	0.733 (0.308)

a HRV: heart rate variability.

b FRF: frequency response function.

c IR: impulse response.

d Performance metrics using combined feature sets (HRV+FRF and HRV+IR), compared to the individual IR feature set, for each classification model (LR, SVM linear, and SVM RBF) using NM undersampling.

e AUC-ROC: area under the receiver operating characteristic curve.

f LR: logistic regression.

g SVM: support vector machine.

h RBF: radial basis function.

**Table 7.** Comparison of performance metrics for combined feature sets (HRV^a^+FRF^b^, HRV+IR^c^) and individual IR set, using Synthetic Minority Oversampling Technique (SMOTE)–balanced data^d^.

Feature set and classifier	Accuracy, mean (SD)	Precision, mean (SD)	Recall, mean (SD)	Specificity, mean (SD)	*F*_1_-score, mean (SD)	AUC-ROC^e^, mean (SD)
HRV+FRF						
LR^f^	0.421 (0.183)	0.422 (0.137)	0.550 (0.411)	0.350 (0.253)	0.444 (0.220)	0.446 (0.156)
SVM^g^ linear	0.586 (0.078)	0.553 (0.077)	0.900 (0.137)	0.283 (0.046)	0.680 (0.073)	0.567 (0.231)
SVM RBF^h^	0.586 (0.192)	0.583 (0.373)	0.483 (0.291)	0.683 (0.207)	0.646 (0.105)	0.600 (0.279)
HRV+IR						
LR	0.671 (0.139)	0.650 (0.137)	0.733 (0.181)	0.617 (0.112)	0.686 (0.146)	0.688 (0.201)
SVM linear	0.589 (0.200)	0.581 (0.180)	0.900 (0.137)	0.317 (0.335)	0.690 (0.135)	0.729 (0.116)
SVM RBF	0.700 (0.128)	0.783 (0.217)	0.683 (0.207)	0.750 (0.306)	0.691 (0.097)	0.742 (0.139)
IR						
LR	0.529 (0.156)	0.587 (0.250)	0.600 (0.379)	0.517 (0.291)	0.520 (0.190)	0.629 (0.193)
SVM linear	0.611 (0.062)	0.569 (0.041)	0.950 (0.112)	0.267 (0.181)	0.708 (0.038)	0.771 (0.062)
SVM RBF	0.532 (0.088)	0.434 (0.247)	0.617 (0.439)	0.417 (0.328)	0.615 (0.146)	0.692 (0.216)

a HRV: heart rate variability.

b FRF: frequency response function.

c IR: impulse response.

d Performance metrics using combined feature sets (HRV+FRF and HRV+IR), compared to the individual IR feature set, for each classification model (LR, SVM linear, and SVM RBF) using SMOTE oversampling.

e AUC-ROC: area under the receiver operating characteristic curve.

f LR: logistic regression.

g SVM: support vector machine.

h RBF: radial basis function.

In NM balanced datasets, the combined HRV+FRF feature set yielded higher values than the individual IR feature set in accuracy (0.830, SD 0.172 vs 0.770, SD 0.179), precision (0.800, SD 0.183 vs 0.783, SD 0.217), and *F*_1_-score (0.853, SD 0.145 vs 0.798, SD 0.140) for the SVM RBF classifier. HRV+FRF also produced higher metric values than HRV alone across all metrics for SVM RBF (accuracy: 0.830, SD 0.172 vs 0.730, SD 0.192; precision: 0.800, SD 0.183 vs 0.683, SD 0.207; and *F*_1_-score: 0.853, SD 0.145 vs 0.765, SD 0.190). Among classifiers, SVM RBF exhibited some of the strongest observed performance with HRV+FRF, while SVM linear showed comparable patterns with both HRV+FRF and HRV+IR.

For SMOTE-processed data, the HRV+IR combined feature set yielded higher values than the individual IR feature set in most metrics, particularly in accuracy (SVM RBF: 0.700, SD 0.128 vs 0.532, SD 0.088), precision (0.783, SD 0.217 vs 0.434, SD 0.247), and AUC-ROC (0.742, SD 0.139 vs 0.692, SD 0.216). However, IR alone retained slightly better recall (0.950, SD 0.112 vs 0.900, SD 0.137) and *F*_1_-score (0.708, SD 0.038 vs 0.690, SD 0.135) with the SVM linear classifier. Within this SMOTE-balanced setting, the SVM RBF model showed some of the strongest performance patterns when paired with HRV+IR, while SVM linear also performed well, maintaining a high recall (0.900, SD 0.137) and a comparable *F*_1_-score (0.690, SD 0.135), indicating effective classification sensitivity. The LR model produced moderate metrics with HRV+IR (accuracy: 0.671, SD 0.139; precision: 0.650, SD 0.137; AUC-ROC: 0.688, SD 0.201), highlighting the use of this combined feature set even for simpler models.

In summary, the combined feature sets improved overall classification performance in the SMOTE-balanced dataset, and HRV+IR generally produced comparatively strong results across models. Among the classifiers evaluated, SVM RBF tended to show some of the higher metric values when paired with this feature set.

### Summary of Performance Findings

Across classifiers and balancing conditions, the IR feature set often showed comparatively higher performance as an individual feature set, particularly in terms of recall and *F*_1_-score with SVM linear. Combining IR with HRV offered performance improvements in the SMOTE-balanced scenario and more modest gains with NM. In general, combined feature sets tended to show enhanced performance relative to the unbalanced datasets, particularly when SMOTE was used.

SMOTE tended to increase recall and overall sensitivity, especially for SVM linear, whereas NM produced higher specificity and *F*_1_-scores. These patterns suggest that the choice of sampling technique should be guided by the classification goals and the clinical importance of false positives versus false negatives.

No feature set consistently outperformed others across all metrics or balancing strategies. For instance, the combined HRV+FRF feature set performed well with NM, while HRV+IR showed comparatively better performance under SMOTE. The IR feature set remained competitive as a standalone option, showing similar sensitivity-based performance to HRV+FRF. Under NM, HRV+FRF achieved slightly higher accuracy, specificity, *F*_1_-score, and AUC-ROC than IR alone, while under SMOTE, HRV+IR showed improvements in accuracy (0.168), precision (0.349), specificity (0.333), and AUC-ROC (0.050) relative to IR. IR alone retained slightly higher recall (0.050) and *F*_1_-score (0.018) in that setting, without added feature complexity.

Although combined feature sets offered incremental benefits in several cases, selecting among them should weigh these gains against increased model complexity and limited sample size. For applications where accuracy and precision are emphasized, the HRV+IR feature set under SMOTE may warrant further investigation. For settings prioritizing simplicity or sensitivity to potential T2DM cases, the IR feature set under NM may remain a practical alternative. Overall, these findings represent hypothesis-generating patterns that may guide future analysis in larger and more diverse cohorts.

## Discussion

### Overview of Findings

This study investigated the effectiveness of various feature sets, classification models, and data balancing techniques for distinguishing individuals with and without T2DM. The findings highlight the strengths and limitations of different approaches while examining the discriminative value of HRV, FRF, and IR metrics. To the best of our knowledge, this is the first study to evaluate these complementary domains of autonomic and cardiorespiratory regulation within a ML framework in the context of T2DM. By systematically assessing individual and combined feature sets, this study provides exploratory insight into physiologically grounded patterns that may differentiate cardiorespiratory regulation between individuals with and without T2DM, supporting future investigations in larger and independently validated cohorts.

### Principal Results

#### Classification Performance Across Feature Sets

Across individual feature sets, IR function metrics tended to show comparatively higher predictive performance for distinguishing individuals with and without T2DM. This pattern may reflect IR metric’s ability to capture causal, time-domain characteristics of RCC, which could be sensitive to subtle regulatory differences associated with diabetes. Prior work in related fields, such as obstructive sleep apnea [21], has demonstrated the value of causal analyses of cardiorespiratory interactions, and the present findings extend that insight in the context of diabetes. To the authors’ knowledge, this is the first study to evaluate IR metrics in this setting, providing preliminary evidence that motivates further validation in larger and clinically characterized cohorts.

Similarly, Marmarelis et al [39] used IR estimation methods to model the causal, directional influences of arterial blood pressure and CO_2_ fluctuations (inputs) on cerebral blood velocity (CBV, output). In particular, from the estimated IRs, they derived principal dynamic modes—a data-based modeling technique that decomposes these responses into key dynamic components—identifying significant reductions in principal dynamic modes gain (indicating weakened regulatory responses) for both arterial blood pressure-to-CBV and CO_2_-to-CBV pathways in patients with T2DM compared to controls. This approach enabled the creation of a composite diagnostic index with an AUC of 0.78 for differentiating T2DM from controls, underscoring the value of integrating IR-based directional modeling and noncausal measures in detecting subtle physiological impairments.

In our study, when feature sets were combined (HRV+FRF or HRV+IR), we observed improved or comparable performance compared to IR alone. These patterns likely reflect the complementary physiological information captured by the different domains: HRV summarizes the overall frequency-domain structure of cardiac variability, FRF metrics describe frequency-specific transfer properties of respiratory–cardiac interactions, and IR metrics quantify causal dynamic responsiveness to respiratory inputs.

Prior work also supports the integrative use of multivariate autonomic and cardiorespiratory descriptors. Emerging evidence suggests that metrics targeting specific physiological pathways can reveal regulatory differences not captured by global HRV indices. For example, reductions in respiratory–cardiac interactions have been suggested as early indicators of impaired autonomic regulation in type 2 diabetes [40]. Similarly, baroreflex measures derived from causal, model-based approaches have outperformed traditional spontaneous indices in predicting clinical outcomes and identifying autonomic impairment in patient cohorts [41]. Together, these findings highlight the value of model-based approaches for characterizing pathway-specific physiological regulation.

Taken together, these findings suggest that HRV, FRF, and IR metrics probe different aspects of cardiorespiratory autonomic regulation and may offer complementary perspectives when assessed individually and in combination. Nonetheless, given the exploratory nature of the present analysis and the modest sample size, improvements with combined feature sets may also partly reflect increased feature dimensionality rather than purely additive physiological contributions.

It is important to emphasize that the classifiers in this study distinguish diabetes status, not clinically diagnosed autonomic dysfunction. Therefore, the observed differences likely reflect diabetes-related physiological alterations that may involve autonomic components, but our models cannot be interpreted as detecting or predicting autonomic impairment at the individual level.

#### Influence of Balancing Techniques on Performance

Balancing techniques significantly influenced classification performance. NM consistently improved specificity and *F*_1_-scores, refining class distinction by retaining T2DM (the majority class) instances closest to the opposite class. This is particularly important for imbalanced datasets where negative class identification is often challenging.

In contrast, SMOTE enhanced sensitivity (recall) for some classifiers (especially SVM linear), by generating synthetic samples for the minority class (controls), though specificity gains were less consistent. SMOTE’s synthetic samples are not tailored to emphasize the decision boundary and may lead to overlap between classes and, consequently, a reduced precision in distinguishing control cases.

Similarly, in a study to assess the efficacy of different ML models and balancing techniques for diabetes diagnosis using an imbalanced multiclass dataset (with class 0: nondiabetic, class 1: prediabetic as minorities, and class 2: diabetic as the majority) [42], the authors found that the overall recall (macro-averaged across classes) for SVM (linear) and SVM (RBF) improved with SMOTE oversampling compared to NM undersampling, though per-class results were mixed (eg, lower class 1 recall for SVM RBF with SMOTE, despite gains in classes 0 and 2). In contrast, when LR was used as the classifier, recall was substantially higher using NM balancing compared to SMOTE, with NM outperforming SMOTE across all classes—substantial gains in class 1 (prediabetic, the minority class, +0.40) and smaller improvements in class 0 (+0.14) and class 2 (diabetic, the majority class, +0.05)—suggesting NM is more effective at balancing the dataset for this classifier, particularly for the minority class. Specificity was not evaluated in this study.

These conceptual differences highlight the importance of selecting a balancing technique aligned with the predictive goals. The choice depends on prioritizing specificity (NM) or sensitivity (SMOTE), guided by the clinical implications of false positives versus false negatives. In clinical screening contexts, where the aim is often to identify individuals at higher metabolic or cardiovascular risk, minimizing false negatives (maximizing recall/sensitivity) is typically more important, as missed cases may delay further evaluation and preventive care. Because SMOTE tended to improve recall across feature sets and classifiers, it may be better aligned with population-level screening or risk-stratification workflows where sensitivity is prioritized.

#### Classifier-Specific Observations

While more complex models like SVM RBF occasionally achieved higher performance, linear SVM and LR models offered comparable results, especially when paired with IR features. This suggests that even simpler, more interpretable models can perform competitively when the feature set is physiologically meaningful—an important consideration for clinical adoption.

#### Implications for Understanding T2DM Classification

The findings of this study suggest that dynamic, causal features, particularly those derived from IR metrics, may capture physiologically meaningful differences in cardiorespiratory regulation between individuals with and without T2DM. IR measures quantify the responsiveness of RRI to respiratory perturbations, which may reflect aspects of autonomic adaptability not fully represented by traditional HRV and FRF metrics. In this context, reductions in IR measures may indicate changes in RCC that warrant further investigation in larger cohorts.

Across classifiers and balancing strategies, the generally strong performance of the IR feature set indicates that modeling causal, time-domain dynamics can provide useful discriminatory information when exploring how T2DM relates to autonomic and cardiorespiratory regulation. The integration of static (HRV and FRF) and dynamic (IR) metrics offers a preliminary multivariate perspective on physiological regulation in T2DM, supporting the hypothesis that complementary domains may capture different aspects of diabetes-related physiological differences.

Because this analysis is exploratory and based on a modest sample size, these interpretations should be viewed as hypothesis-generating. Future studies with well-defined autonomic phenotyping will be necessary to clarify the extent to which these physiological patterns reflect autonomic regulation, microvascular alterations, metabolic factors, or other T2DM-related mechanisms.

### Limitations

While this study provides valuable insights into the classification of T2DM using various feature sets, classifiers, and data balancing techniques, several limitations should be noted.

A primary limitation is the relatively modest sample size of the PhysioNet datasets used, particularly for ML applications in which multiple feature sets and classifiers are evaluated. This limited sample size reduces statistical power, increases fold-to-fold variability in cross-validation, and heightens the risk of overfitting—despite our use of feature standardization, correlation-based feature reduction, and 5-fold cross-validation to mitigate these issues. Consequently, the generalizability of the findings to broader and more heterogeneous T2DM populations is uncertain. Larger, more diverse datasets will be essential to validate the models, confirm the stability of the feature sets, and establish their applicability across different demographic and clinical subgroups. For these reasons, the conclusions of the present study should be interpreted as preliminary and exploratory.

Protocol-related differences across datasets represent an additional limitation. While the protocol for the Cerebral Perfusion and Cognitive Decline in Type 2 Diabetes dataset [17] included paced breathing, the Cerebral Vasoregulation in Diabetes dataset [16] did not involve controlled respiratory conditions. These differences may influence respiratory patterns, autonomic engagement, and cardiorespiratory coupling dynamics, potentially affecting the FRF and IR estimates used in this study. As such, part of the observed variability may reflect protocol-specific physiological factors rather than group differences alone. Future studies using harmonized experimental designs will be important to isolate and interpret these effects.

A further methodological limitation relates to the implementation of the class-balancing procedures. In this exploratory analysis, NM undersampling and SMOTE oversampling were applied to the full usable dataset prior to generating the 5-fold cross-validation partitions, rather than separately within each training fold. This approach enabled direct comparison of balancing strategies under fixed-class distributions, but it also means that, particularly for SMOTE, synthetic samples were generated using neighborhood information from the entire dataset and could subsequently appear in both training and test folds. This introduces a degree of information leakage and may partially contribute to the variability observed in some performance metrics. Such variability is also expected given the modest sample size, where each fold contains relatively few test samples, making sensitivity, specificity, and *F*_1_-score more sensitive to fold composition. For these reasons, the performance estimates should be viewed as preliminary. Future studies with larger datasets will be able to implement fold-wise balancing and preprocessing to avoid this issue and obtain more stable and generalizable results.

A related limitation concerns the correlation-based feature filtering step. To maintain consistent feature definitions across all model configurations, correlation filtering (threshold =0.8) was applied once to the full usable dataset rather than separately within each cross-validation fold. This choice avoided the instability and inconsistency that fold-wise feature selection would likely introduce in a small dataset, but it also means that correlation structure from the entire dataset—including samples later assigned to the test folds—contributed to the filtering process. As a result, this preprocessing decision introduces a potential source of information leakage and could lead to mildly optimistic performance estimates. Future work with larger cohorts will enable fold-wise filtering and more sophisticated assessments of feature redundancy (eg, variance inflation factor, mutual information, principal component analysis–based methods) while preserving model stability.

A key challenge of incorporating IR metrics is the need for respiration measurements, which introduces an extra layer of complexity to the practical implementation of the clinical testing protocol. This additional signal channel can complicate data acquisition and processing, potentially limiting the feasibility of the approach in resource-constrained settings. However, recent technological advancements have mitigated some of these challenges by introducing standalone instrumentation systems that are both portable and low-cost, capable of simultaneously measuring ECG and respiration signals. Examples of such systems include the Protocentral tinyECG module (Protocentral), which uses the MAX30001 chip (Maxim Integrated/Analog Devices) to integrate biopotential and bioimpedance channels for ECG and respiration measurements, and the Equivital LifeMonitor (Equivital), which provides clinical-grade ECG and breathing rate measurements via impedance. These systems offer user-friendly interfaces and affordability, potentially reducing barriers to adoption in clinical practice.

It should be acknowledged that BMI is a known modulator of autonomic regulation. Because the usable dataset was small and BMI was strongly collinear with T2DM status, adjusting for BMI (eg, via analysis of covariance or covariate-adjusted modeling) would have further reduced statistical power and produced potentially unstable estimates. The goal of this analysis was to examine the mechanistic and discriminative value of the physiological features (HRV, FRF, and IR) rather than to isolate covariate-adjusted effects. Therefore, BMI was not included as a covariate. We acknowledge that part of the observed group differences may reflect the physiological influence of adiposity rather than diabetes-specific autonomic dysfunction alone. Future studies with larger and more diverse cohorts will be needed to disentangle the independent contributions of adiposity and diabetes.

Moreover, because HRV, FRF, and IR feature domains are all derived from cardiac timing data—and FRF and IR additionally incorporate respiratory inputs—some degree of redundancy among features is expected. To reduce multicollinearity, we applied correlation-based filtering (threshold =0.8) across all feature configurations, including the standalone and combined feature sets. Although HRV, FRF, and IR capture different but related aspects of autonomic and cardiorespiratory regulation, residual redundancy may remain, and some performance gains from combined feature sets may partly reflect increased dimensionality rather than strictly complementary physiological information. Larger datasets will be needed to more clearly differentiate the unique versus overlapping contributions of these domains.

Additionally, the study’s reliance on specific classifiers (LR, SVM linear, and SVM RBF) and balancing techniques (NM and SMOTE) may restrict generalizability to other ML frameworks or preprocessing pipelines. Furthermore, while combined feature sets occasionally improved performance, these gains were modest relative to the added model complexity. Future studies should evaluate the trade-offs between feature aggregation, interpretability, and computational efficiency.

### Comparison With Prior Work

As stated by the American Diabetes Association [43], CAN is asymptomatic in its early stages and detected only by HRV calculated from recording an ECG either during a shift from a seated to a standing posture or during a 1‒2 minute deep breathing test in the doctor’s office, both of which require patient cooperation. Using time, frequency, and nonlinear HRV indices from both resting and orthostatic challenge data, Rathod et al [44] showed that a classification and regression tree model showed an accuracy of 0.840, sensitivity of 0.895, a specificity of 0.667, and an AUC of 0.78 compared to resting HRV alone with 0.751 accuracy, 0.864 sensitivity, 0.392 specificity, with an AUC of 0.63 for differentiating autonomic dysfunction in nondiabetic control and T2DM.

In our study, the HRV feature set obtained from sitting data showed comparatively lower performance for distinguishing individuals with and without T2DM, also suggesting that sitting HRV alone may lack the granularity needed to capture physiological differences associated with diabetes. IR metrics, which capture the dynamic influence of respiration on RRI, frequently demonstrated stronger discriminative performance than HRV and FRF features within our dataset. For example, the IR feature set obtained from sitting data showed an accuracy of 0.770, sensitivity of 0.900, specificity of 0.633, and an AUC of 0.700 (LR using NM balancing) for differentiating T2DM from controls, values comparable to those reported by Rathod et al [44] despite using resting data alone. These findings suggest that causal, time-domain representations of respiratory-cardiac interactions may capture physiological distinctions between T2DM and control groups that are not fully reflected in resting HRV or noncausal FRF metrics.

Our modeling framework also differs from Rathod et al [44] in its use of LR and SVM classifiers rather than a CART model. CART provides explicit, rule-based decision pathways that are easily interpretable in clinical settings. LR offers coefficient-based interpretability, whereas SVMs rely on margin-based discrimination that emphasizes classification boundaries rather than direct feature-level explanations. Therefore, these models represent alternative analytical strategies, each with distinct strengths in terms of transparency and decision structure.

Finally, although IR- and FRF-based metrics may provide physiologically motivated insights into respiratory–cardiac regulation, further work incorporating validated autonomic outcomes will be required to determine their relevance in assessing autonomic impairment in diabetes.

While our approach leverages physiologically interpretable IR metrics for passive monitoring, other studies have explored automated diabetes detection using deep learning models applied to ECG-derived signals, though often at the cost of interpretability. Swapna et al [45] used a hybrid deep neural network combining a convolutional neural network–long short-term memory (CNN–LSTM) using RRI time series (derived from ECG signals) as input, achieving 95.1% accuracy in diabetes detection. These RRIs represent raw HRV data without specific feature extraction. In a subsequent study, Swapna et al [46] integrated an SVM classifier following the CNN–LSTM architecture, improving accuracy to 95.7%.

However, the absence of additional performance metrics, such as precision, sensitivity, and specificity, obscures the models’ ability to minimize false positives and false negatives. Furthermore, deep learning approaches like CNN–LSTM are noninterpretable, offering limited insight into which HRV features drive classification outcomes. In contrast, our study examined feature domains—spectral HRV, FRF, and IR metrics—chosen for their physiological grounding in autonomic and cardiorespiratory regulation. These features provide mechanistic insight by characterizing overall variability (HRV), frequency-domain transfer properties (FRF), and causal dynamic responsiveness to respiratory inputs (IR). However, while IR-derived measures such as IR magnitude, DG, and $t_{char}$ are physiologically interpretable within a systems-modeling framework, they require specialized technical understanding. The value of these metrics lies in their potential to complement traditional HRV-based assessments by probing different regulatory pathways. Future work incorporating validated autonomic phenotyping will be necessary to determine whether these physiologically motivated descriptors can be translated into clinically interpretable or actionable tools.

### Conclusion

This study highlights the potential value of dynamic cardiorespiratory metrics—particularly IR features—for distinguishing individuals with and without T2DM. By modeling the causal, time-domain characteristics of RCC, IR metrics frequently demonstrated comparatively strong and physiologically interpretable discriminative performance, complementing the information provided by traditional HRV and noncausal FRF measures.

Given the modest sample size and the exploratory nature of the analysis, these findings should be interpreted as preliminary. Performance estimates may be affected by dataset-specific characteristics, limited statistical power, and the risk of overfitting, and therefore may not generalize to broader populations.

Taken together, the results suggest that systems-based cardiorespiratory features—spanning variability measures, frequency-domain transfer properties, and causal dynamic responses—capture physiological differences associated with T2DM that merit further investigation. Future studies using larger and more diverse cohorts with validated autonomic phenotyping will be essential to clarify how these features relate to clinically meaningful autonomic regulation and to evaluate their broader translational relevance. Such work will help determine the extent to which these physiological domains contribute to our understanding of diabetes-related regulatory changes.

## Supplementary material

10.2196/82084 Multimedia Appendix 1 Frequency response function and impulse response methodologies.

## Abbreviations

AUC-ROC: area under the receiver operating characteristic curve
BIDMC: Beth Israel Deaconess Medical Center
CAN: cardiovascular autonomic neuropathy
CARTs: cardiovascular autonomic reflex tests
CBV: cerebral blood velocity
CNN–LSTM: convolutional neural network–long short-term memory
ECG: electrocardiogram
FRF: frequency response function
HF: high-frequency
HRV: heart rate variability
ILV: instantaneous lung volume
IR: impulse response
IRB: institutional review board
LF: low-frequency
LR: logistic regression
ML: machine learning
NM: NearMiss-1
RBF: radial basis function
RCC: respiratory-cardiac coupling
RRI: R-to-R interval
SMOTE: Synthetic Minority Oversampling Technique
SVM: support vector machine
T2DM: type 2 diabetes mellitus
