# Supplementary material for: Cardiorespiratory Markers of Type 2 Diabetes: Machine Learning–Based Analysis
Source: JMIR Diabetes. 2026 Feb 23;11:e82084. doi: 10.2196/82084 (PMC12928690; doi:10.2196/82084)
Supplement: Multimedia Appendix 1 [file diabetes-v11-e82084-s001.doc]

**Online supplement**

**Frequency response function (FRF)**

The frequency response function (FRF) is a systems-level approach designed to mathematically characterize the dynamic relationship between an input
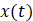
 and an output
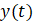
. Here,
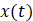
 and
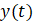
 represent fluctuations of each variable around its mean.

In the context of cardiovascular variability, the FRF
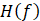
 quantifies how oscillations in one cardiovascular or cardiorespiratory variable (e.g. respiration) affect oscillations in another (e.g. heart rate or R-R interval). Formally,
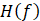
 is defined in the frequency domain as:


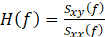
 , (1)

where:

-
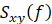
 is the cross-spectral density between input
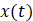
 and output
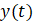
, quantifying how much power (or variance) at each frequency is shared between the two signals, thereby measuring the strength of their relationship at different frequencies;

-
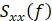
 is the auto-spectral density of the input, representing how the power (or variance) of
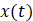
 is distributed across different frequencies.

The magnitude
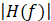
 indicates the gain, or the degree of amplification or attenuation, of the input’s effect on the output at each frequency, normalized by the spectral characteristics of the respiratory pattern (as measured by
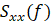
) [1], while its phase determines the phase shift introduced by the system. In this study, we focus on the magnitude of the FRF.

For example, if the input represents oscillations in instantaneous lung volume
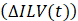
 and the output corresponds to oscillations in the R-R interval signal
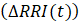
, the frequency response function (or transfer function)
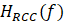
 between input
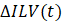
 and output
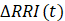
 characterizes the respiratory-cardiac coupling (RCC) mechanism, with higher gains indicating stronger modulation of heart rate by respiratory oscillations [2]. This coupling involves vagal feedback from lung stretch receptors, respiratory modulation of cardiovagal neural output, effects of intrathoracic pressure on cardiopulmonary receptors, and direct mechanical compression and stretching of the sinoatrial node [3].

Thus, the FRF between pairs of input-output physiological variables typically represents the aggregate behavior of multiple physiological mechanisms governing their interrelationship [4]. Compared to univariate heart rate variability (HRV) analysis, the FRF
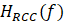
 normalizes the heart rate response to the spectral characteristics of the respiratory pattern, mitigating the dependency of HRV on breathing rate—a known limitation of univariate HRV analysis [5, 6]. This normalization enhances the interpretability of autonomic contributions to heart rate fluctuations, providing a more mechanistic understanding of regulatory dynamics.

To quantify these effects, we estimated the FRF between input
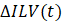
 and output
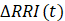
 and computed the gain
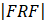
, defined as the area under the curve of the magnitude of the transfer function. Specifically, we extracted the low-frequency (
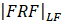
) and high-frequency (
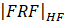
) components as features for the FRF approach.

Several studies have applied the FRF to investigate cardiovascular and autonomic regulation. For example, Berger et al. [2] utilized the FRF to analyze the dynamic response of the sinoatrial node to vagal and sympathetic stimuli, offering insights into autonomic modulation of heart rate. Saul et al. [7] applied the FRF to characterize respiratory sinus arrhythmia (RSA), demonstrating how respiratory oscillations drive heart rate variability. Additionally, Robbe et al. [8] and Pitzalis et al. [9] used the FRF to estimate baroreflex sensitivity from spontaneous fluctuations in systolic arterial pressure and heart rate, providing a non-invasive measure of this critical regulatory mechanism.

A key limitation of the FRF technique is its assumption of a unidirectional relationship between input and output, making it applicable only to open-loop systems. However, cardiovascular control involves complex closed-loop interactions, where feedback mechanisms influence the observed dynamics. As a result, the FRF conflates contributions from both direct and feedback mechanisms, as it cannot distinguish causal directions or separate overlapping influences [10].

**Impulse response (IR) metrics**

To address the non-causal limitations of the FRF, we implemented a causal time-domain multivariate analysis using system identification techniques based on the impulse response function. This systems-level approach, adapted from systems engineering, involves constructing a mathematical model that captures the underlying dynamics between an input and an output based on measured data [11].

The estimated impulse response (IR) of two related time series—an input
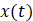
 and an output
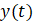
—defines a mathematical model of the physiological mechanisms governing their relationship [12, 13]. Unlike the FRF, this time-domain approach allows for a representation where the output
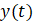
 explicitly depends on present and past values of the input
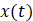
, but not on future values, while also incorporating delays into the model. As a result, the impulse response function provides a mathematical framework to effectively “open the loop”, thereby separating the direct pathway linking
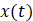
 to
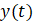
 from the feedback components acting in the opposite direction [12, 13].

For example, the impulse response function between respiratory oscillations
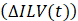
 and R-R interval variations
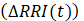
captures the dynamics of heart rate regulation via the respiratory-cardiac coupling (RCC) mechanism, independently of blood pressure effects on heart rate. The latter can be investigated by estimating the impulse response function between systolic blood pressure (SBP) fluctuations and RRI oscillations, modeling the dynamics of heart rate regulation via the baroreflex, independently of respiratory influences on heart rate.

From each estimated impulse response, we derived the following compact quantitative descriptors as features for machine learning classifiers:

**(1) Impulse Response Magnitude (IRM)**: This metric represents the gain of the impulse response, defined as the difference between its maximum and minimum values. IRM reflects the strength of the immediate response of the output to a unit impulse in the input. Belozeroff et al. [14] demonstrated that the baroreflex gain (IRM of IR_ABR_, or IR between ΔSBP as input and ΔRRI as output) increased following long-term continuous positive airway pressure (CPAP) therapy in patients with obstructive sleep apnea (OSA), suggesting an increase in vagal activity with consistent CPAP use;

**(2) Dynamic Gain (DG)**: This measure corresponds to the average magnitude of the causal transfer function,
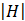
, computed from the Fourier transform of the estimated IR. It indicates how strongly the input influences the output across different frequency ranges. For instance, Chaicharn et al. [15] found that the low-frequency component of the dynamic gain (
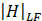
)—derived from the baroreflex impulse response (IR_ABR_)—was lower in OSA patients than in controls, indicating baroreflex sensitivity dysfunction. Additionally, Belozeroff et al. [16] reported that both IRM and DG of the respiratory-cardiac coupling impulse response (IR_RCC_) were significantly lower in OSA patients than in controls, reflecting reduced vagal and increased sympathetic modulations in OSA;

**(3) Latency (*L*)**: This parameter quantifies the time delay between the onset of a stimulus at the input and the actual response in the output. Jo et al. [12] found that latency associated with the baroreflex mechanism (*L*_ABR_) was significantly prolonged in OSA patients compared to healthy individuals, indicating slower autonomic reflex responses;

**(4) Characteristic time (*t*_char_)**: This metric captures the time window within which the majority of the impulse response occurs. A longer *t*_char_ suggests a more prolonged response, while a shorter value indicates a quicker return to baseline. It serves as a summary measure of the temporal evolution of the response, condensing its time course into a single descriptive value.

These compact quantitative descriptors were used as features in our IR-based classification approach.

# References

| [1] | M. C. Khoo, T. S. Kim and R. B. Berry, "Spectral indices of cardiac autonomic function in obstructive sleep apnea," *Sleep,* vol. 22, no. 4, p. 443–451, Jun 1999. https://doi.org/10.1093/sleep/22.4.443 |
| --- | --- |
| [2] | R. D. Berger, J. P. Saul and R. J. Cohen, "Transfer function analysis of autonomic regulation. Part I. Canine atrial rate response," *American Journal of Physiology-Heart and Circulatory Physiology,* vol. 256, no. 1 Pt 2, p. H142–H152, 1989. https://doi.org/10.1152/ajpheart.1989.256.1.H142 |
| [3] | M. C. K. Khoo, "Modeling of autonomic control in sleep-disordered breathing," *Cardiovascular Engineering,* vol. 8, no. 1, pp. 30-41, 2008. https://doi.org/10.1007/s10558-007-9041-9 |
| [4] | J. Batzel, G. Baselli, R. Mukkamala and K. H. Chon, "Modelling and disentangling physiological mechanisms: linear and nonlinear identification techniques for analysis of cardiovascular regulation," *Philosophical Transactions, Series A, Mathematical, Physical, and Engineering Sciences,* vol. 367, no. 1892, pp. 1377-91, 13 April 2009. https://doi.org/10.1098/rsta.2008.0266 |
| [5] | G. E. Billman, "The LF/HF ratio does not accurately measure cardiac sympatho-vagal balance," *Frontiers in Physiology,* vol. 4, no. Article 26, Feb 2013. https://doi.org/10.3389/fphys.2013.00026 |
| [6] | D. S. Quintana, G. A. Alvares and J. A. J. Heathers, "Guidelines for Reporting Articles on Psychiatry and Heart rate variability (GRAPH): recommendations to advance research communication," *Translational Psychiatry,* vol. 6, no. e803, 10 May 2016. https://doi.org/10.1038/tp.2016.73 |
| [7] | J. P. Saul, R. D. Berger, M. H. Chen and R. J. Cohen, "Transfer function analysis of autonomic regulation. Part II. Respiratory sinus arrhythmia," *American Journal of Physiology-Heart and Circulatory Physiology,* vol. 256, no. 1 Pt 2, p. H153–H161, Jan 1989. https://doi.org/10.1152/ajpheart.1989.256.1.H153 |
| [8] | H. W. Robbe, L. J. Mulder, H. Rüddel, W. A. Langewitz, J. B. Veldman and G. Mulder, "Assessment of baroreceptor reflex sensitivity by means of spectral analysis," *Hypertension,* vol. 1, pp. 538-43, 1987. https://doi.org/10.1161/01.hyp.10.5.538 |
| [9] | M. V. Pitzalis, F. Mastropasqua, F. Massari, A. Passantino, R. Colombo, A. Mannarini, C. Forleo and P. Rizzon, "Effect of respiratory rate on the relationships between RR interval and systolic blood pressure fluctuations: a frequency-dependent phenomenon," *Cardiovascular Research,* vol. 38, no. 2, p. 332–9, May 1998. https://doi.org/10.1016/s0008-6363(98)00029-7 |
| [10] | L. Faes, A. Porta, R. Cucino, S. Cerutti, R. Antolini and G. Nollo, "Causal transfer function analysis to describe closed loop interactions between cardiovascular and cardiorespiratory variability signals," *Biological Cybernetics,* vol. 90, no. 6, p. 390–399, Jun 2004. https://doi.org/10.1007/s00422-004-0488-0 |
| [11] | K. H. Chon, R. Mukkamala, K. Toska, T. J. Mullen, A. A. Armoundas and R. J. Cohen, "Linear and nonlinear system identification of autonomic heart-rate modulation," *IEEE Engineering in Medicine and Biology Magazine,* vol. 16, no. 5, p. 96–105, Sep-Oct 1997. https://doi.org/10.1109/51.620500 |
| [12] | J. A. Jo, A. Blasi, E. Valladares, R. Juarez, A. Baydur and M. C. K. Khoo, "Model-based assessment of autonomic control in obstructive sleep apnea syndrome during sleep," *American Journal of Respiratory and Critical Care Medicine,* vol. 167, no. 2, pp. 128-36, Feb 2003. https://doi.org/10.1093/sleep/26.1.65 |
| [13] | M. C. K. Khoo, Physiological control systems: analysis, simulation, and estimation, 2nd ed., John Wiley & Sons, 2018. |
| [14] | V. Belozeroff, R. B. Berry, C. S. H. Sassoon and M. C. K. Khoo, "Effects of CPAP therapy on cardiovascular variability in obstructive sleep apnea: a closed-loop analysis," *American Journal of Physiology-Heart and Circulatory Physiology,* vol. 282, no. 1, p. H110–H121, 2002. https://doi.org/10.1152/ajpheart.2002.282.1.H110 |
| [15] | J. Chaicharn, Z. Lin, M. L. Chen, S. L. D. Ward, T. Keens and M. C. K. Khoo, "Model based assessment of cardiovascular autonomic control in children with obstructive sleep apnea," *Sleep,* vol. 32, no. 7, p. 927–38, Jul 2009. https://doi.org/10.1093/sleep/32.7.927 |
| [16] | V. Belozeroff, R. B. Berry and M. C. K. Khoo, "Model-based assessment of autonomic control in obstructive sleep apnea syndrome," *Sleep,* vol. 26, no. 1, pp. 65-73, 2003. https://doi.org/10.1093/sleep/26.1.65 |
